# Edge-AI Instrumentation Framework for Multimodal Biometric Sensing in Active Aging Environments

**DOI:** 10.3390/s26165072

**Published:** 2026-08-10

**Authors:** Teresa Guarda, Washington Torres-Guin, Jairo R. Coronado-Hernández, Arnulfo Alanis

**Affiliations:** 1 Faculty of Systems and Telecommunications, Universidad Estatal Península Santa Elena, Santa Elena 240204, Ecuador; 2 Department of Productivity and Innovation, Universidad de la Costa, Barranquilla 080002, Colombia; 3 Systems and Computer Department, National Technology of México, Campus Tijuana, Calzada del Tecnológico S/N, Fraccionamiento Tomas Aquino, Tijuana C.P. 22414, Mexico

**Keywords:** edge AI, instrumentation framework, multimodal biometric sensing, wearable sensors, ambient sensors, active aging, older adults, signal acquisition, sensor calibration, signal quality, temporal synchronization, edge computing, real-time monitoring, smart environments, health monitoring

## Abstract

Population aging has increased the need for continuous, non-invasive, and context-aware monitoring systems capable of supporting autonomy, safety, and early intervention in daily living environments. Multimodal biometric sensing offers an important technical basis for this purpose, as it combines physiological, motion-related, and environmental signals to provide a more complete view of older adults’ functional and health-related conditions. However, many existing solutions remain fragmented, device-dependent, and insufficiently connected to core instrumentation requirements, including signal quality, sensor calibration, temporal synchronization, latency, energy consumption, interoperability, reliability, and data privacy. This article proposes an Edge-AI instrumentation framework for multimodal biometric sensing in active aging environments, supported by a structured analysis of recent literature on wearable, ambient, and context-aware sensing systems. The framework integrates wearable, ambient, and context-aware sensors with local processing capabilities to support signal acquisition, preprocessing, quality control, feature extraction, anomaly detection, and decision support close to the data source. By placing Edge AI within the instrumentation pipeline, the proposed framework identifies design requirements that may help reduce response time, limit unnecessary transmission of sensitive biometric data, and improve feasibility in home-based and assisted-living contexts. These expected benefits, however, require empirical testing through future prototype implementation and real-world evaluation. The article also defines a validation-oriented perspective for sensor-based active aging systems, covering technical, operational, and human-centered dimensions such as measurement accuracy, signal robustness, usability, privacy preservation, interoperability, reproducibility, energy efficiency, and system scalability. The proposed framework is intended to support the design, comparison, and validation of more reliable, interpretable, and reproducible sensor-based monitoring systems, while offering a structured basis for prototype development and future real-world evaluation in active aging environments.

## 1. Introduction

Population aging is reshaping health, care, and social support systems worldwide. According to the World Health Organization, by 2030, one in six people will be aged 60 years or over, and the number of people in this age group is expected to reach 2.1 billion by 2050 [1]. This demographic transition has increased the need for technologies that can support autonomy, safety, and early intervention in daily living environments, particularly as older adults wish to remain active and independent for longer. In this context, active aging is not limited to the absence of disease. It also involves maintaining functional ability, mobility, social participation, and quality of life in environments that are able to respond to gradual changes in physical, physiological, and behavioral conditions [2,3].

Sensor-based monitoring systems have become central to this challenge. Wearable, ambient, and context-aware sensors can collect physiological, motion-related, and environmental data continuously or semi-continuously, offering information that is difficult to obtain through episodic clinical assessment alone [4,5]. Wearable devices may capture heart rate, oxygen saturation, electrodermal activity, gait, posture, and physical activity patterns. Ambient sensors, including pressure sensors, motion detectors, acoustic sensors, depth cameras, and environmental sensors, can provide complementary information about daily routines, movement in the home, risk situations, and interaction with the surrounding environment [3,6]. When these data streams are combined, multimodal biometric sensing can offer a more complete view of functional status and health-related changes in older adults [2].

In this article, multimodal biometric sensing is understood in a broad instrumentation sense rather than as a narrow reference to identity recognition or physiological biometrics alone. It refers to the combined acquisition and interpretation of physiological, motion-related, behavioral, and environmental signals that can characterize functional status, health-related changes, activity patterns, and risk situations in older adults. This use of the term is appropriate for active aging environments because mobility, gait, posture, activity routines, inactivity, room transitions, and contextual environmental information may be as relevant as direct physiological measurements for assessing autonomy, safety, and functional change.

Despite this potential, the design of sensor-based systems for active aging still faces important technical and methodological limitations. Many solutions remain device-specific, application-specific, or dependent on isolated sensing modalities. In several cases, insufficient attention is given to core instrumentation requirements, such as signal acquisition quality, calibration, noise reduction, temporal synchronization, data loss, sensor drift, measurement uncertainty, latency, and reproducibility [4,5,7]. These aspects are not secondary details. They determine whether a monitoring system can produce reliable data under real-world conditions, where older adults move naturally, devices may be worn inconsistently, home environments vary, and signals are affected by motion artifacts, placement errors, battery limitations, and network constraints [4,6].

The integration of Edge Artificial Intelligence offers a relevant response to some of these limitations. By processing data close to the source, edge-based systems can reduce dependence on remote cloud infrastructures, lower communication latency, support faster detection of relevant events, and limit the transmission of sensitive biometric data [2,7]. In active aging environments, this is particularly important because risk-related events, such as falls, abnormal inactivity, physiological instability, or changes in daily routines, may require timely interpretation [7]. Edge AI also creates opportunities for local feature extraction, anomaly detection, adaptive filtering, and context-aware decision support. However, the use of AI at the edge also introduces constraints related to computational capacity, energy consumption, model robustness, update mechanisms, and explainable system behavior [2,7].

For this reason, the development of active aging monitoring systems should not be approached only as a problem of data collection or algorithmic classification. It should also be understood as an instrumentation problem. A reliable system must define what is measured, how it is measured, under which conditions the measurement remains valid, how signals from different sensors are synchronized, and how uncertainty is handled across the sensing and processing pipeline [4,6,7]. This perspective is especially relevant for multimodal systems, where physiological, behavioral, and environmental signals may differ in frequency, format, quality, and clinical or functional meaning. Without a clear instrumentation framework, the integration of multiple sensors and Edge AI may produce technically complex systems that are difficult to validate, compare, reproduce, or use in real care contexts [2,5].

This article addresses this gap by proposing an Edge-AI instrumentation framework for multimodal biometric sensing in active aging environments. The framework connects sensor acquisition, signal preprocessing and quality control, local feature extraction, edge-based inference, communication, data governance, and decision support within a structured measurement-oriented architecture. Its purpose is to define how heterogeneous sensing components and Edge-AI processes can be organized as a coherent instrumentation pipeline. This perspective is important because the reliability of active aging monitoring depends not only on the availability of sensors or algorithms but also on the conditions under which data are acquired, synchronized, processed, interpreted, protected, and validated. In doing so, the article contributes to the design of monitoring systems that are more reproducible, privacy-aware, interoperable, and suitable for real-world active aging contexts.

The main contributions of this article are threefold. First, it provides a structured analysis of recent research on wearable, ambient, contactless, and context-aware sensing technologies used in active aging environments. Second, it identifies the main instrumentation requirements that affect the reliability of sensor-based systems, including signal quality, calibration, synchronization, latency, energy consumption, interoperability, privacy, security, and reproducibility. Third, it proposes a conceptual layered Edge-AI instrumentation framework that organizes multimodal sensing, signal preparation, local inference, data governance, decision support, and validation as a coherent pipeline for future prototype development, empirical testing, and real-world evaluation. Unlike previous Edge-AI or IoT-based monitoring architectures that mainly emphasize data processing, connectivity, or application-specific detection tasks, the proposed framework is explicitly instrumentation-oriented. It integrates signal acquisition, calibration, quality control, temporal synchronization, local inference, data governance, decision support, and validation within a single layered pipeline for active aging environments.

The remainder of this article is organized as follows. Section 2 presents the methodological approach used to support the structured literature analysis and the development of the proposed framework. Section 3 reports the main findings related to sensing modalities, processing approaches, application contexts, and validation practices. Section 4 discusses the findings, presents the proposed Edge-AI instrumentation framework, and outlines implications and limitations. Finally, Section 5 concludes the article.

## 2. Materials and Methods

This study was designed as a framework-development article supported by a structured literature analysis. The literature analysis followed the PRISMA 2020 statement to ensure a transparent and reproducible process for identifying, screening, selecting, and synthesizing relevant studies [8]. The methodological design was also aligned with the requirements of Sensors, particularly regarding the availability of search records, screening decisions, data extraction procedures, analytical criteria, and reporting transparency.

The literature analysis focused on studies related to multimodal biometric sensing, wearable and ambient sensors, Edge AI, local processing, and active aging environments. Since the topic combines sensor instrumentation, intelligent data processing, health monitoring, and smart environments, the study did not aim to perform a statistical meta-analysis. Instead, it used a structured descriptive and thematic synthesis to identify sensing modalities, technical architectures, instrumentation challenges, validation practices, and design requirements for the proposed Edge-AI instrumentation framework [8].

### 2.1. Study Design and Analytical Protocol

The study followed a framework-development approach supported by a predefined analytical protocol for the structured literature analysis. The protocol established the research questions, information sources, search strings, inclusion and exclusion criteria, screening procedure, data extraction fields, quality assessment criteria, and synthesis strategy before the full screening process was conducted. This decision was taken to reduce selection bias, improve traceability, and support the reproducibility of the literature-based framework development process [8].

The structured literature analysis had two complementary aims. The first aim was to identify and characterize recent studies that use sensor-based systems for biometric, functional, behavioral, or environmental monitoring in active aging, smart-home, assisted-living, or older adult care contexts. The second aim was to extract technical evidence relevant to the design of an Edge-AI instrumentation framework, with special attention to signal acquisition, sensor calibration, signal quality, synchronization, latency, energy consumption, interoperability, privacy, reliability, and reproducibility [4,5,7].

A structured literature analysis was considered appropriate because the evidence in this field is technically heterogeneous. Studies differ in sensor type, measured signals, target population, hardware platform, processing architecture, validation strategy, and application context. For this reason, the analysis was structured to support both descriptive mapping and conceptual integration, providing the basis for the proposed instrumentation framework [8].

### 2.2. Research Questions

The structured literature analysis was guided by five research questions:

**RQ1.**  *What types of wearable, ambient, and context-aware sensors are used for biometric and functional monitoring in active aging environments?*

**RQ2.**  *Which physiological, motion-related, behavioral, and environmental signals are most frequently collected and analyzed in these systems?*

**RQ3.**  *How are Edge AI, edge computing, or local processing approaches used to support real-time monitoring, anomaly detection, and decision support?*

**RQ4.**  *What instrumentation challenges are reported in relation to signal quality, calibration, synchronization, latency, energy consumption, reliability, interoperability, privacy, and reproducibility?*

**RQ5.**  *Which validation criteria are used to assess the technical and practical performance of sensor-based systems for active aging?*

These research questions were defined to keep the analysis centered on sensors, measurement systems, and instrumentation requirements, rather than treating the topic only as a general application of AI in healthcare [2,4,6].

### 2.3. Information Sources and Search Strategy

The literature search was conducted in scientific databases commonly used in engineering, sensor systems, computer science, biomedical engineering, health technology, and smart environments research. The information sources were Scopus, Web of Science, IEEE Xplore, PubMed, ScienceDirect, SpringerLink, and MDPI, with particular attention to Sensors because of its direct relevance to sensor devices, instrumentation, IoT, wearable technologies, intelligent sensing, and health monitoring systems.

Because the article focuses on sensor-based instrumentation, multimodal sensing, wearable and ambient sensors, and Edge-AI-enabled monitoring, Sensors was searched as a journal-specific source in addition to multidisciplinary databases. This decision was justified by the journal’s explicit scope in sensor science and technology, wearable sensors, IoT sensing systems, non-invasive assistive technologies, and reproducible sensor-based research. To reduce source concentration bias, the search also included multidisciplinary and publisher-based databases covering engineering, computer science, biomedical engineering, and health technology.

The search covered studies published between 2020 and 2026. Only documents written in English were considered. The search strategy was organized around three thematic blocks: sensor technologies, edge-based processing, and active aging contexts. The first block included terms related to biometric sensors, wearable sensors, ambient sensors, multimodal sensors, physiological monitoring, motion sensing, and signal acquisition. The second block included terms related to Edge AI, edge computing, edge intelligence, embedded AI, local processing, and IoT-based analytics. The third block included terms related to active aging, older adults, elderly people, assisted living, smart homes, independent living, home monitoring, and aging environments [2,4,5,7].

The main search string was defined as follows:

(“biometric sensors” OR “wearable sensors” OR “ambient sensors” OR “multimodal sensors” OR “physiological sensors”) AND (“edge AI” OR “edge computing” OR “edge intelligence” OR “embedded AI” OR “local processing”) AND (“active aging” OR “older adults” OR “elderly” OR “assisted living” OR “smart home” OR “independent living”)

A complementary search string was used to strengthen the instrumentation focus:

(“sensor instrumentation” OR “measurement system” OR “signal acquisition” OR “signal quality” OR “calibration” OR “synchronization”) AND (“wearable sensors” OR “biometric sensors” OR “ambient sensors” OR “multimodal sensing”) AND (“older adults” OR “active aging” OR “assisted living” OR “smart home”)

A third search string focused on validation and performance:

(“wearable sensors” OR “biometric sensors” OR “ambient sensors” OR “multimodal sensors”)

AND (“validation” OR “calibration” OR “accuracy” OR “reliability” OR “latency” OR “energy consumption”) AND (“older adults” OR “elderly” OR “smart home” OR “health monitoring”)

The search strings were adapted to the syntax of each database while preserving the same conceptual structure. Boolean operators, quotation marks, field restrictions, filters, search dates, and the number of records retrieved from each source were documented in the search log included in the Supplementary Material [8].

The literature search was conducted on 25 April 2026. For each database or source group, the adapted search string, search date, applied filters, and number of records retained after filtering were recorded in the study database. The 110 candidate records correspond to the records retained after applying the predefined topic, publication period, language, document-type, and relevance filters at the database/source level. This clarification is important because the 110 records do not represent all raw search hits returned by the databases but the candidate records retained after the initial database/source-level filtering process. The complete search log, including source-specific search information, filters, retained records, and coding notes, is provided in Supplementary File S1.

Table 1 summarizes the database/source-level distribution of the 110 candidate records retained after filtering and before duplicate removal.

### 2.4. Eligibility, Screening, and Study Selection

The eligibility criteria were defined to identify studies that provide technical and methodological evidence relevant to sensor-based monitoring systems and to the development of the proposed framework for active aging environments. Studies were included if they met the following criteria: publication between 2020 and 2026; English language; focus on wearable sensors, ambient sensors, biometric sensors, multimodal sensing, or sensor-based monitoring systems; relation to older adults, active aging, assisted living, smart homes, independent living, health monitoring, or functional monitoring; and inclusion of technical information about sensors, data acquisition, signal processing, system architecture, Edge AI, IoT, local processing, or validation. Peer-reviewed journal articles, review papers, and highly relevant conference papers were considered [8].

Studies were excluded if they were not related to sensors, sensing systems, or instrumentation; focused only on clinical intervention without a sensor-based monitoring component; presented AI models without describing the sensor data or measurement context; were opinion papers, editorials, commentaries, or non-peer-reviewed documents; were not available in full text; were duplicates; were not written in English; or fell outside the selected publication period.

All records identified through database searching were exported to the study database. Duplicate records were removed using a combination of title, author names, DOI, and publication metadata. The remaining records were screened by title, abstract, and keywords. Studies that clearly fell outside the scope of the study were excluded at this stage. Records that appeared relevant or uncertain were assessed through full-text assessment [8].

The screening and eligibility assessment were conducted by two reviewers. Disagreements or uncertain cases were discussed until consensus was reached. When necessary, a third author was consulted to support the final decision. This procedure was adopted to reduce individual selection bias and improve the transparency and reproducibility of the study selection process. Formal inter-rater agreement statistics were not calculated; this limitation is acknowledged in Section 4.6.

During full-text assessment, each study was checked against the inclusion and exclusion criteria. Reasons for exclusion were recorded, including lack of sensor focus, absence of technical detail, unrelated target population, no Edge AI or local processing component, insufficient instrumentation relevance, or unavailable full text. The final set of included studies formed the basis for the descriptive analysis, thematic synthesis, and development of the proposed framework.

The database search identified 110 candidate records. Six duplicate records were removed before screening, leaving 104 unique records for title, abstract, and keyword screening. During screening, 31 records were separated as background/contextual support because they contributed to the conceptual, methodological, or technological framing of the study but were not eligible for the final synthesis corpus. Therefore, 73 reports were sought for retrieval. All 73 reports were retrieved and assessed for full-text eligibility. After full-text assessment and final reference cross-checking, 35 reports were excluded and 38 studies were included in the final synthesis corpus. The reconciled flow was therefore as follows: 110 identified records − 6 duplicates = 104 screened records; 104 screened records − 31 background/contextual records = 73 reports sought for retrieval; 73 reports sought − 0 reports not retrieved = 73 reports assessed for eligibility; and 73 reports assessed − 35 reports excluded = 38 studies included in the final synthesis.

The 31 background/contextual records were not included in the final synthesis corpus and were not used to calculate the frequencies reported in Section 3. They were used only to support the conceptual, methodological, and technological framing of the manuscript, including the definition of search terms, interpretation of broader research trends, and contextualization of active aging, Edge AI, and sensor-based monitoring. All quantitative results and synthesis tables are based exclusively on the 38 studies included in the final synthesis corpus.

To avoid ambiguity, background/contextual records were used only in sections requiring conceptual or contextual framing, such as in Section 1 and parts Section 4. They were not used to support the frequency counts, category distributions, synthesis tables, or statements reported in Section 3 derived from the final synthesis corpus. The reference cross-checking process was revised to ensure that citations used in Section 3 correspond to records included in the final synthesis corpus.

**Figure 1:**
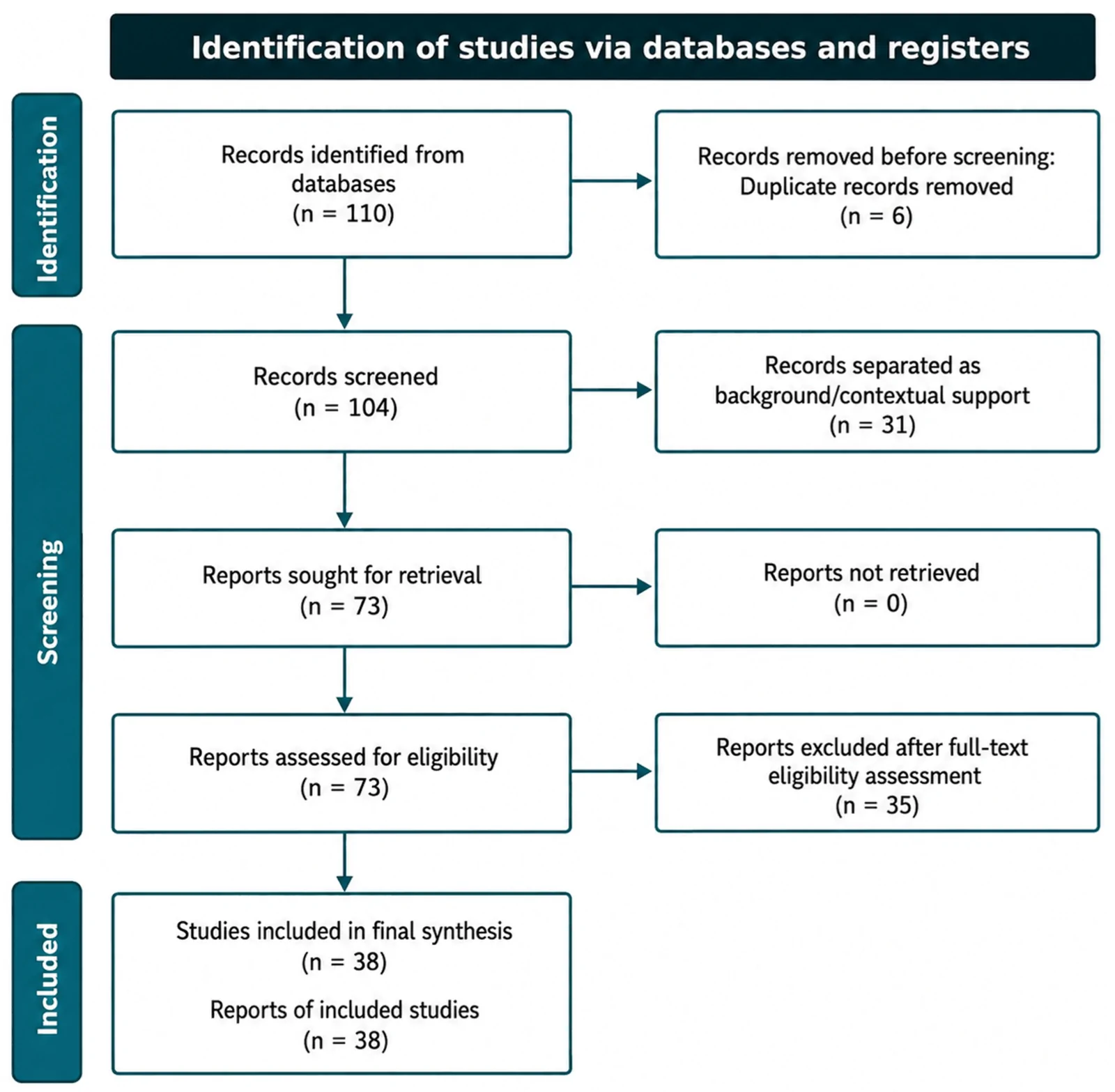
PRISMA 2020 flow diagram of the study selection process.

The complete identification, screening, eligibility, and inclusion process is presented in Figure 1. The full search log, duplicate removal decisions, screening decisions, exclusion reasons, and coding decisions are provided in Supplementary File S1.

The diagram summarizes the identification, duplicate removal, screening, retrieval, eligibility assessment, and final inclusion stages used to define the synthesis corpus. A total of 110 candidate records were identified, 6 duplicates were removed, and 104 records were screened. Of these, 31 records were used only as background or contextual support, while 73 reports were assessed for eligibility. After full-text assessment and final reference cross-checking, 35 reports were excluded and 38 studies were included in the final synthesis.

### 2.5. Data Extraction, Quality Assessment, and Synthesis

A structured data extraction matrix was used to collect comparable information from each included study. The extracted fields included author(s), year of publication, title, source, country or study context when available, study type, target population, application context, sensor type, sensing modality, measured signals, device or hardware platform, data acquisition method, sampling frequency when reported, preprocessing method, AI or machine learning approach, edge, cloud, or hybrid architecture, communication technology, validation method, performance metrics, reported limitations, instrumentation challenges, and relevance to the proposed framework [8].

The quality and technical relevance of the included studies were assessed using a checklist adapted to sensor-based and instrumentation-oriented research. The checklist considered whether each study clearly reported the sensor type and placement, measured variables, acquisition conditions, preprocessing procedures, AI or signal processing methods, validation strategy, performance metrics, limitations, reproducibility details, and relevance to active aging or older adult monitoring. Each criterion was scored using a three-level scale: 1.0 for full compliance, 0.5 for partial compliance, and 0.0 for non-compliance [8].

Studies were not excluded solely because of lower reporting quality unless missing information prevented meaningful interpretation of the sensing or instrumentation contribution. However, quality assessment results were considered when discussing the strength, limitations, and reproducibility of the evidence.

The synthesis combined descriptive and thematic analysis. Descriptive analysis was used to summarize publication trends, sensor types, sensing modalities, measured signals, application contexts, processing architectures, and validation methods. Thematic analysis was used to identify recurrent instrumentation challenges and design requirements. These dimensions supported the development of the proposed Edge-AI instrumentation framework, which organizes the main technical requirements for reliable multimodal biometric sensing in active aging environments [8].

Bibliographic records, screening decisions, data extraction, coding, quality-assessment scores, and descriptive tabulations were managed using Microsoft Excel for Microsoft 2013 (Microsoft Corporation, Redmond, WA, USA). No automated screening or statistical-analysis software was used.

### 2.6. Reproducibility and Ethics

To support transparency and reproducibility, the complete study database is provided as Supplementary File S1. This file includes the search log, database-specific search strings, search dates, number of records retained after filtering from each source, duplicate removal information, title and abstract screening decisions, full-text assessment decisions, exclusion reasons, PRISMA counts, quality assessment scores, and the final data extraction table.

No chemicals, reagents, commercial cell lines, biological samples, commercial materials, devices, or instruments were directly used in this study. All references to sensors, devices, instruments, and hardware platforms concern technologies reported in the studies included in the structured literature analysis.

No large experimental dataset was generated in this study. The data supporting the study consist of bibliographic records and coded information extracted from previously published studies. These data are made available through the Supplementary Material submitted with the manuscript.

This study is based on a structured analysis of published literature and the development of a conceptual instrumentation framework. It did not involve human participants, animals, clinical intervention, or the collection of personal data. Therefore, ethical approval was not required.

## 3. Results

This section presents the findings from the 38 studies included in the final synthesis corpus. The results are organized according to the research questions defined in Section 2. Section 3.1 presents the general characteristics of the included studies, while Section 3.2 reports the quality assessment results. RQ1 and RQ2 are addressed in Section 3.3, which reports sensing modalities and measured signals. RQ3 is addressed in Section 3.4, which examines Edge AI and processing architectures. RQ4 is addressed in Section 3.6, which reports instrumentation and validation challenges. RQ5 is addressed across Section 3.5 and Section 3.6, where application contexts and validation practices are discussed. This structure allows the analysis to move from descriptive mapping and quality appraisal to technical interpretation, providing the empirical basis for the Edge-AI instrumentation framework proposed in Section 4.3.

### 3.1. Descriptive Overview of the Included Study Corpus

The included studies show a clear concentration of recent research on sensor-based monitoring systems for older adults, smart homes, assisted living, and active aging environments. Since the final synthesis corpus covers studies published between 2021 and 2025, it reflects a recent body of work marked by the growing use of wearable devices, ambient sensing infrastructures, IoT-based architectures, and Edge-AI-supported monitoring approaches.

The temporal distribution confirms that the field has expanded strongly in the most recent years. Of the 38 studies included in the final synthesis corpus, 15 were published in 2025, 13 in 2024, and 7 in 2023. Together, these three years account for 35 studies, corresponding to 92.1% of the final synthesis corpus. Earlier years were less represented, with 2 studies published in 2021 and 1 study published in 2022. No study from 2026 was retained in the final synthesis corpus.

The corpus includes different types of studies, including experimental articles, prototype-based studies, system architecture papers, dataset-based evaluations, systematic reviews, scoping reviews, broader review articles, and pilot or validation-focused studies. For reporting purposes, document types were grouped into three broader categories. Research articles included records coded as “Article” in Supplementary File S1 (*n* = 26). Reviews and review-like studies included systematic reviews (*n* = 4), scoping reviews (*n* = 3), review articles (*n* = 2), and systematic review and meta-analysis studies (*n* = 1), corresponding to 10 studies in total. Pilot or validation-focused studies included validation studies (*n* = 1) and pilot studies (*n* = 1), corresponding to 2 studies in total. This grouping explains how the detailed document-type coding reported in Supplementary File S1 maps onto the summarized categories used in the manuscript.

A relevant feature of the corpus is the strong presence of studies published in Sensors. Of the 38 studies included in the final synthesis, 26 were published in Sensors, corresponding to 68.4% of the final corpus. The remaining 12 studies were drawn from other journals and sources, including IEEE, JMIR, Frontiers, Elsevier, Springer-related sources, ACM, Diagnostics, and conference-related sources. This distribution remains methodologically coherent because the article focuses on sensor science, instrumentation, wearable and ambient sensors, IoT sensing systems, and reproducible sensor-based research [5,7,9,10,11].

Table 2 summarizes the main descriptive characteristics of the included studies, including publication period, source distribution, document type, and general analytical dimensions. This overview provides a structured basis for interpreting the more detailed results presented in the following subsections, particularly those related to sensing modalities, processing architectures, application contexts, and validation challenges. The complete list of records, coding decisions, and study-level classifications is provided in Supplementary File S1.

### 3.2. Quality Assessment of the Included Studies

The quality assessment showed that most included studies provided sufficient information on sensor type, application context, and performance metrics. However, reporting was less consistent for sensor placement, acquisition conditions, calibration procedures, sampling frequency, preprocessing details, synchronization, and reproducibility materials. These results were relevant for the development of the proposed framework because they showed that the main limitation of the literature is not the absence of sensing or AI solutions, but the incomplete reporting of the measurement and processing pipeline. The full quality assessment scores are provided in Supplementary File S1.

Table 3 summarizes the main quality assessment findings and their influence on the development of the proposed framework.

### 3.3. Sensing Modalities and Measured Signals

The included studies show that sensor-based monitoring in active aging environments is built around several complementary sensing modalities. Because many studies combine more than one type of sensor or analytical purpose, the categories reported in this subsection are not mutually exclusive. Consistent with the definition introduced in Section 1, biometric sensing is used here in a broad instrumentation sense, covering physiological, motion-related, behavioral, and environmental signals that support the interpretation of functional and health-related conditions in older adults.

The most frequent group was related to motion, mobility, activities of daily living (ADL), and fall-related sensing. This category appeared in 34 studies, reflecting the central role of movement analysis in active aging research. These studies used sensors to monitor falls, fall risk, gait, balance, physical activity, mobility loss, frailty-related changes, human activity recognition, and daily living patterns. This emphasis is consistent with the practical needs of active aging environments, where mobility decline, abnormal inactivity, and falls are among the most relevant risks for older adults [11,12,13,14,15].

Wearable and body-worn sensing was also highly represented, appearing in 25 studies. These studies used or discussed devices such as inertial measurement units, accelerometers, gyroscopes, wrist-worn devices, smart watches, wearable motion sensors, smart textiles, and other body-worn technologies. Wearable sensors are particularly useful because they provide direct information about movement, posture, gait, activity levels, and, in some cases, physiological parameters. However, the reviewed literature also reports recurrent concerns about device placement, comfort, battery life, motion artifacts, user adherence, and long-term use in real-world conditions [5,16,17,18,19].

Ambient and non-wearable sensing appeared in 16 studies. This group included binary sensors, passive infrared sensors, smart-home sensors, pressure sensors, environmental sensors, non-wearable monitoring infrastructures, and proximity-based sensing devices. These technologies are relevant for older adults because they reduce the need for continuous user interaction with a device. They are especially useful in home-based monitoring, where daily routines, room transitions, inactivity, and environmental context can provide relevant information about functional status and risk situations [7,9,10,20,21].

Contactless sensing technologies, including radar, Wi-Fi sensing, depth cameras, vision-based systems, and related approaches, appeared in 7 studies. These technologies provide an important alternative when wearable devices are uncomfortable, inconsistently used, or unsuitable for long-term monitoring. Contactless sensing can also support privacy-sensitive applications when properly designed, particularly in fall detection, posture monitoring, indoor movement analysis, and smart-home contexts [16,21,22,23,24,25,26].

Multimodal or sensor-fusion systems were explicitly identified in 4 studies. These systems are particularly relevant because they combine wearable, ambient, proximity-based, robotic, or IoT-based data streams to improve contextual interpretation in elderly care and home health monitoring. Although this number is smaller than expected given the theme of the present article, several studies combined modalities indirectly through IoT architectures, smart-home systems, or hybrid wearable and ambient monitoring. This suggests that the field still lacks mature and reproducible approaches for integrating heterogeneous signals into a unified instrumentation pipeline [27,28,29].

One study focused primarily on physiological sensing, such as heart rate, respiratory activity, oxygen saturation, temperature, or other physiological signals. This indicates that, within the current corpus, active aging monitoring is still more strongly oriented toward motion, activity, fall detection, and functional assessment than toward continuous physiological monitoring. This is an important finding for the proposed framework because it shows the need to connect motion-based, physiological, behavioral, and environmental sensing more explicitly in future multimodal systems, as shown in Table 4.

Table 4 summarizes the sensing modalities identified in the included studies. Since several studies used more than one sensing modality, the totals exceed the number of included studies.

### 3.4. Edge AI and Processing Architectures

The included studies show increasing interest in local and intelligent processing, although the maturity of Edge AI varies considerably across the corpus. The most frequent processing category was AI or machine-learning-based analysis, identified in 29 studies. These studies used or discussed methods such as classical machine learning, deep learning, anomaly detection, activity recognition models, fall detection algorithms, prediction models, and classification approaches applied to sensor data. AI-based analysis was especially common in fall detection, gait classification, activity recognition, anomaly detection, and risk prediction [12,13,30,31,32].

To avoid overlap between categories, processing architectures were defined according to the dominant processing location and system function. Edge/local/embedded processing refers to systems in which relevant preprocessing, feature extraction, inference, or anomaly detection is performed close to the sensor, gateway, or local device. IoT/cloud/smart-home architectures refer to systems primarily designed around sensor connectivity, remote platforms, cloud storage, or smart-home integration. Hybrid architectures refer to systems that combine local processing with cloud-based storage, visualization, model updating, or long-term analytics. When a study included more than one architecture, it was coded according to its dominant contribution, while secondary components were recorded in the extraction matrix.

Edge, local, or embedded processing was explicitly present in 4 studies. These studies are particularly relevant to the purpose of this article because they move data processing closer to the sensor or local environment. In active aging contexts, this is important because events such as falls, abnormal inactivity, or physiological instability may require rapid interpretation. Edge processing can reduce latency, decrease dependence on continuous cloud connectivity, and limit the transmission of sensitive biometric or behavioral data [7,33].

IoT, cloud, or smart-home architectures appeared in 7 studies. These systems typically connect multiple sensors, gateways, home infrastructures, and remote platforms. They provide scalability and integration, but they also raise concerns about latency, data transfer, interoperability, privacy, and long-term maintenance. Hybrid architectures, combining local processing and cloud-based support, were also present in several studies, although they were not always described in enough technical detail to support full reproducibility [29,34,35,36].

A smaller group of studies focused on signal processing, statistical analysis, optimization, or graph-based analytics. These approaches are important because sensor-based monitoring does not depend only on AI classification. It also depends on preprocessing, filtering, segmentation, feature extraction, uncertainty handling, and signal-quality control. In several studies, however, these steps were not reported with sufficient detail, which limits comparison and replication [19,20,37,38,39].

In one study, processing was not central or was not clearly reported. This does not make the study irrelevant, but it indicates a reporting gap. For a journal such as Sensors, where instrumentation, measurement, and reproducibility are central, insufficient description of the processing pipeline reduces the technical value of otherwise relevant studies. Table 5 summarizes the main processing architectures and analytical approaches identified in the corpus.

The table shows the main processing approaches reported in the included studies, including AI/ML-based analysis, edge/local/embedded processing, IoT/cloud/smart-home architectures, signal/statistical/optimization processing, and cases where processing was not central or not clearly reported.

### 3.5. Application Contexts in Active Aging Environments

The included studies addressed several application contexts related to active aging, older adult care, assisted living, and smart environments. As with the sensing categories, these application categories are not mutually exclusive, because many studies addressed more than one monitoring objective.

All studies included in the final synthesis corpus were relevant to active aging or older adult monitoring according to the eligibility criteria. However, the specific application context “older adults/active aging” was explicitly coded in 24 studies, corresponding to 63.2% of the final synthesis corpus. This distinction clarifies that the eligibility scope of the corpus is broader than the individual application-context categories used for frequency coding. This confirms that the corpus is well aligned with the central purpose of the review. These studies focused on supporting autonomy, safety, functional monitoring, independent living, and early detection of changes that may affect older adults’ daily life [3].

Health monitoring and care support appeared in 12 studies, corresponding to 31.6% of the final synthesis corpus. These studies included home health monitoring, assisted-care support, remote monitoring, rehabilitation-related monitoring, and systems intended to support caregivers or health professionals. However, the technical depth varied. Some studies provided detailed information about sensors, signals, architecture, and validation, while others remained closer to conceptual or system-level descriptions [10].

Fall detection and fall-risk assessment were present in 13 studies, corresponding to 34.2% of the final synthesis corpus, making this one of the most developed application domains in the corpus. This strong representation is expected, since falls are a major concern in aging populations and are directly linked to safety, autonomy, hospitalization, and care needs [11,13,15,39,40].

Activity recognition and activities of daily living monitoring appeared in 16 studies, corresponding to 42.1% of the final synthesis corpus. These studies used sensor data to identify daily routines, movement patterns, behavioral changes, and functional activity. ADL monitoring is particularly relevant because active aging does not depend only on detecting acute events; it also requires understanding gradual changes in daily functioning [18,21,24,27,41].

Mobility, gait, balance, and frailty-related monitoring appeared in 10 studies, corresponding to 26.3% of the final synthesis corpus. These topics are important because mobility decline can precede more serious functional deterioration. Sensor-based gait and balance assessment can support early intervention, rehabilitation planning, and fall-risk evaluation. The reviewed studies show that this area uses a wide range of technologies, including wearable inertial sensors, in-shoe sensors, wrist-worn devices, contactless gait systems, and sensor-based functional tests [14,19,37,38,42,43].

Smart-home and assisted-living contexts appeared in 12 studies, corresponding to 31.6% of the final synthesis corpus. These studies provide the environmental setting for many of the technologies analyzed in this study. They also highlight the importance of unobtrusive sensing, interoperability, user acceptance, privacy, and long-term deployment. Smart-home and AAL systems are particularly relevant because they allow monitoring to occur in daily living environments rather than only in clinical or laboratory settings [34,36].

Table 6 summarizes the application-context categories coded in the final synthesis corpus. Categories are not mutually exclusive, and the counts refer to studies coded in each application-context category rather than to the broader eligibility scope of the review.

### 3.6. Instrumentation and Validation Challenges

The structured analysis identified several recurrent instrumentation and validation challenges that directly affect the reliability and practical use of sensor-based systems in active aging environments. These challenges are especially important because the technical success of a monitoring system depends not only on the selected sensors or algorithms, but also on how measurements are acquired, synchronized, processed, validated, interpreted, and protected.

Signal quality was one of the most recurrent concerns. Wearable sensors can be affected by motion artifacts, incorrect placement, poor contact with the body, clothing interference, and inconsistent use. Ambient sensors can be affected by room layout, occlusion, furniture position, environmental noise, lighting conditions, and the presence of multiple occupants. Poor signal quality can lead to false alerts, missed events, and unreliable interpretation of functional, behavioral, or physiological changes [19,37,38,39]. In the coding process, signal quality was identified as relevant across all 38 studies, not because each study addressed it adequately, but because the reliability of data acquisition and interpretation depends on signal quality across wearable, ambient, and contactless monitoring systems. Calibration and measurement consistency were also important concerns. Sensor outputs may change over time because of device drift, battery state, placement variation, environmental conditions, or hardware differences. In multimodal systems, calibration becomes more complex because different devices may measure different variables at different sampling rates and with different levels of precision. The included literature shows that many studies report accuracy or classification performance, but fewer describe calibration procedures, acquisition conditions, or uncertainty handling in enough detail to support replication [16,19,37]. Calibration was explicitly identified as a relevant instrumentation challenge in 8 studies, corresponding to 21.1% of the final synthesis corpus. This lower frequency supports the interpretation that calibration remains less consistently reported than broader concerns such as signal quality, energy consumption, interoperability, and reproducibility. Synchronization is a central issue in multimodal monitoring. Physiological, motion-related, behavioral, and environmental signals often operate on different time scales. If these data streams are not temporally aligned, the interpretation of events may become unreliable. For instance, a sudden movement detected by a wearable sensor may require confirmation from an ambient sensor, location signal, or contextual variable [27,28,29].

Latency and energy consumption were particularly relevant in Edge-AI and wearable systems. In fall detection, abnormal inactivity, or other risk-related events, delayed interpretation can reduce the practical value of the system. At the same time, local processing requires computational resources, which may reduce battery life in wearable or embedded devices. This creates a trade-off between accuracy, response time, computational load, and device autonomy [7,33].

Interoperability remains a structural challenge. Many systems combine sensors, devices, protocols, gateways, cloud platforms, and software tools that were not designed to operate together. This limits scalability and makes it difficult to compare systems across studies. Interoperability problems also affect long-term maintenance, especially in smart-home and assisted-living environments where devices may need to be replaced, updated, or integrated with external care platforms [34,35,36]. Interoperability was identified as relevant in 35 studies, corresponding to 92.1% of the final synthesis corpus. This high frequency reflects the broad dependence of active aging monitoring systems on multi-device, multi-platform, gateway-based, cloud-connected, or care-integrated infrastructures. It should not be interpreted as evidence that these studies implemented mature or standardized interoperability solutions. Privacy and security were also central to the included literature. Active aging systems may collect sensitive information about movement, health status, daily routines, room occupancy, sleep, inactivity, frailty, and behavioral change. Edge AI can reduce the transmission of raw data, but it does not eliminate the need for encryption, access control, de-identification, secure storage, and clear governance of biometric and behavioral data [7,10].

Finally, reproducibility was a recurring limitation. Several studies did not report enough detail about sensor placement, acquisition settings, sampling frequency, preprocessing, validation procedures, or implementation constraints. This limits the ability of other researchers to replicate, compare, validate, or extend the systems. For this reason, reproducibility should be treated as a core instrumentation requirement rather than as an optional reporting detail [10,19,20].

Table 7 summarizes the main technical challenges affecting the reliability, reproducibility, and practical deployment of sensor-based monitoring systems in active aging environments. In this table, a supporting study refers to a study in which the challenge was identified as relevant to the sensing, processing, deployment, validation, or interpretation of the monitoring system. This coding does not mean that the study fully addressed, solved, or validated the challenge. For example, signal quality and interoperability were frequently relevant across the corpus, but their methodological treatment was often incomplete. For this reason, they are interpreted as recurrent instrumentation concerns rather than as maturely resolved aspects of the literature.

### 3.7. Synthesis of Findings

The results show that active aging monitoring is moving toward more integrated sensing ecosystems, but the field remains uneven in terms of instrumentation maturity. Wearable sensors are widely used because they provide direct information about movement, posture, activity, and, in some cases, physiological state. Ambient and contactless sensors complement this information by capturing routines, room-level behavior, inactivity, and context without requiring continuous device use. However, explicitly multimodal and sensor-fusion systems are still less common than expected, which indicates that integration remains a technical and methodological challenge.

The findings also show that AI and machine learning are already central to sensor-based monitoring, especially in fall detection, activity recognition, mobility assessment, and anomaly detection. Nevertheless, Edge AI is less consistently represented than general AI/ML approaches. This indicates that many studies still focus on classification performance rather than on full edge-based instrumentation pipelines that include acquisition, preprocessing, quality control, local inference, latency control, energy constraints, communication, privacy, and validation.

This limited number of explicitly implemented Edge-AI and multimodal systems is an important limitation of the available evidence. For this reason, the proposed framework should be interpreted as an evidence-informed, conceptual, and instrumentation-oriented framework, rather than as an empirically validated architecture. Its purpose is to organize the technical requirements that current studies report incompletely and to guide future implementation and validation efforts.

Across the corpus, the main gap is not the lack of sensors or algorithms. Rather, the gap lies in the limited integration of sensing, measurement quality, local processing, validation, privacy, and reproducibility into a coherent system-level framework. This finding supports the need for the Edge-AI instrumentation framework proposed in Section 4.3. The framework is therefore grounded in the evidence that reliable active aging systems require more than isolated devices or AI models; they require a structured instrumentation pipeline capable of connecting multimodal sensing, signal processing, quality control, edge inference, communication, data governance, decision support, and validation.

### 3.8. Research Question Closure

To close the structured analysis explicitly, Table 8 maps each research question to the corresponding synthesis result. This table also clarifies the distinction between sensing modalities, measured signal groups, processing architectures, instrumentation challenges, and validation criteria. The counts refer to studies in the final synthesis corpus and should be interpreted as study-level coded categories rather than mutually exclusive variables.

For RQ2, the measured signal-group counts are derived from the same study-level modality and application-context coding used in Table 4 and Table 6. They should therefore be interpreted as a proxy signal-group summary rather than as an independent signal-level recoding.

## 4. Discussion

The results of this study show that sensor-based monitoring for active aging has become a technically mature but still fragmented research area. The synthesis corpus confirms the strong presence of wearable sensors, ambient sensing infrastructures, contactless monitoring technologies, IoT-based architectures, and AI-supported analytical methods. However, the findings also show that many systems are still designed around specific devices, isolated use cases, or algorithmic performance metrics, with less attention to the full instrumentation pipeline required for reliable deployment in real-world aging environments.

### 4.1. Interpretation of the Main Findings

The predominance of motion, mobility, ADL, and fall-related sensing indicates that active aging research remains strongly connected to functional monitoring. This is expected, since mobility decline, abnormal inactivity, gait changes, balance impairment, and falls are among the most visible risk indicators in older adults. Wearable sensors are useful in this context because they provide direct information about body movement, posture, gait, and activity levels. However, their practical value depends on consistent use, correct placement, comfort, device autonomy, and robustness against motion artifacts [4,5,16,19].

Ambient and contactless sensing technologies address some of these limitations by reducing the need for continuous device use. Binary sensors, passive sensors, radar, Wi-Fi sensing, depth cameras, and smart-home infrastructures can capture routines, room transitions, inactivity, environmental conditions, and movement patterns in daily living environments. These systems are particularly relevant for long-term monitoring because they are less intrusive and can operate in the background. Nevertheless, they introduce other limitations, including occlusion, room-layout dependency, multi-occupant ambiguity, privacy concerns, and weaker individual-level physiological interpretation [7,9,10,25,44].

A second relevant finding is the gap between general AI/ML use and explicit Edge-AI implementation. Many studies use machine learning or deep learning for fall detection, activity recognition, anomaly detection, and gait classification, but fewer studies describe complete edge-based pipelines. This distinction is important. AI classification alone does not guarantee timely response, privacy protection, low energy consumption, or operational reliability. Edge AI becomes meaningful only when the model is integrated with signal acquisition, preprocessing, quality control, local inference, communication control, latency management, and validation under realistic conditions [2,7,33,45,46].

The analysis also shows that multimodal and sensor-fusion systems are less frequent than expected. Although many studies discuss wearable, ambient, and IoT-based sensing, only a smaller group explicitly integrates heterogeneous data streams into a coherent multimodal monitoring system. This is a relevant limitation because active aging environments are complex. A single sensor rarely captures the full context of an event. For instance, a fall-risk situation may involve gait instability, room location, inactivity, posture, prior movement patterns, and environmental conditions. These elements require temporal alignment, semantic integration, and clear interpretation rules [27,28,29].

### 4.2. Instrumentation Challenges and Research Gaps

The central gap identified in this study is not the absence of sensing technologies. Rather, it is the limited integration of sensing, measurement quality, Edge AI, data governance, validation, and reproducibility within a single instrumentation-oriented design. Several studies report classification accuracy, detection performance, or system feasibility, but fewer provide detailed information about sensor calibration, acquisition conditions, sampling frequency, preprocessing, synchronization, uncertainty, latency, or energy consumption. This weakens comparison across studies and limits the practical transfer of prototypes into real homes, assisted-living facilities, and care environments.

The findings also show that current Edge-AI-based active aging systems remain limited by a gap between experimental performance and real-world readiness. Many studies report promising accuracy values, but fewer address the operational conditions required for deployment, such as long-term sensor stability, calibration drift, missing data, multi-occupant environments, device replacement, model update strategies, user adherence, and integration with care workflows. This gap suggests that future research should move beyond isolated performance evaluation and include deployment-oriented validation protocols.

Signal quality remains one of the most important challenges. Wearable systems are affected by motion artifacts, incorrect placement, inconsistent use, and battery limitations, while ambient systems are affected by occlusion, environmental interference, room layout, and multi-person conditions. These problems can lead to false alarms, missed events, or misleading interpretations. For this reason, signal-quality assessment should be treated as a core design requirement, not only as a preprocessing step [19,37,38,39].

Calibration and synchronization are also essential for multimodal systems. When sensors operate with different sampling rates, locations, formats, and precision levels, their outputs cannot be combined reliably without temporal and measurement alignment. This issue becomes more important when the system combines physiological, behavioral, motion-related, and environmental signals. Without synchronization, sensor fusion may produce technically complex outputs that remain clinically or functionally weak [27,28,29].

Latency and energy consumption are especially relevant in Edge-AI systems. In fall detection, inactivity alerts, and risk-related monitoring, delayed response may reduce the value of the system. At the same time, continuous local processing can increase power consumption and reduce the autonomy of wearable or embedded devices. This creates a design trade-off between model complexity, inference speed, battery life, and reliability. Future systems must therefore be evaluated not only by accuracy but also by response time, computational cost, energy use, and long-term operational stability [7,33,46,47].

Privacy and security also remain central. Active aging systems may collect data about movement, health status, daily routines, room occupancy, sleep, inactivity, and behavioral change. These data are sensitive because they can reveal not only physiological states but also personal habits and vulnerability patterns. Edge AI can reduce the transmission of raw data, but it does not remove the need for encryption, access control, de-identification, secure storage, and clear data-governance rules [2,10,47,48].

### 4.3. Proposed Edge-AI Instrumentation Framework

Based on the findings of this study, this article proposes an Edge-AI instrumentation framework for multimodal biometric sensing in active aging environments. The framework is organized as a layered pipeline that connects sensing, signal preparation, local intelligence, secure communication, and decision-oriented validation. Its purpose is to show how sensor-based monitoring can be designed as an integrated measurement system rather than as a set of isolated devices or AI models. This perspective is important because the reliability of active aging monitoring depends not only on algorithmic accuracy but also on signal acquisition quality, calibration, synchronization, latency control, data governance, energy efficiency, and reproducibility.

Table 9 links the main findings of the structured literature analysis to the corresponding layers of the proposed framework, showing how the identified gaps and instrumentation requirements informed the design of each component.

Table 10 compares the proposed framework with representative existing approaches, highlighting its instrumentation-oriented contribution and the remaining need for empirical validation.

The sensor acquisition layer includes wearable, ambient, contactless, environmental, and context-aware sensors.

The framework is specifically shaped by the needs and constraints of active aging environments. Unlike general-purpose IoT health-monitoring architectures, active aging systems must account for gradual functional decline, fall risk, abnormal inactivity, frailty-related changes, heterogeneous home layouts, variable adherence to wearable devices, privacy expectations, and the need to support autonomy without creating excessive surveillance. These conditions affect how sensing, inference, decision support, and validation should be designed. For example, false-negative and false-positive tolerances in anomaly detection cannot be treated as generic parameters. False negatives may have serious safety implications when falls, prolonged inactivity, or physiological instability are missed, whereas false positives may increase alarm fatigue, reduce user trust, and lead to unnecessary caregiver intervention. For this reason, the proposed framework emphasizes user-specific baselines, context-aware thresholds, feedback-based recalibration, and validation over time.

Its main function is to define what is measured, where it is measured, how frequently it is measured, and under which conditions the measurement can be considered reliable. This layer should include clear information about sensor type, placement, sampling frequency, acquisition environment, calibration requirements, and expected sources of noise.

The signal preprocessing and quality-control layer manages filtering, segmentation, normalization, missing data, artifact reduction, calibration, and synchronization. This layer is essential because Edge-AI models can only produce reliable outputs when the input data are technically valid. For multimodal systems, this layer must also align signals from different sensors and ensure that physiological, motion-related, behavioral, and environmental data are interpreted within the same temporal context.

The Edge-AI inference layer performs local feature extraction, classification, anomaly detection, fall detection, activity recognition, or risk estimation. Its purpose is to process relevant data close to the source, reducing latency and limiting unnecessary transmission of sensitive information. This layer must be designed with attention to model size, computational cost, energy consumption, robustness, inference stability, and update mechanisms.

The communication and data-governance layer manages the transfer of selected information between sensors, gateways, local devices, cloud services, dashboards, or care platforms. It should prioritize privacy-aware communication, secure storage, access control, interoperability, data minimization, and de-identification where appropriate. In active aging environments, this layer is important because monitoring systems may involve older adults, caregivers, clinicians, family members, and technical service providers.

The decision-support and validation layer translates processed information into alerts, indicators, visualizations, or recommendations. This layer should not only report outcomes but also support interpretation. It should include validation criteria such as accuracy, latency, reliability, energy consumption, usability, privacy, security, interoperability, and reproducibility. In this framework, validation is not treated as a final step after system design. It is part of the whole instrumentation process.

The framework also includes a continuous monitoring and feedback loop. This loop indicates that active aging monitoring is not a one-time measurement process. Data collected from daily living environments should support repeated interpretation, model refinement, alert adjustment, system recalibration, and validation over time. In this sense, the framework links real-time monitoring with long-term system improvement. The cross-cutting requirements shown at the bottom of the framework reinforce that privacy, security, interoperability, reliability, reproducibility, and energy efficiency are not isolated concerns. They must be considered across all layers, from sensor se-lection to final decision support.

**Figure 2:**
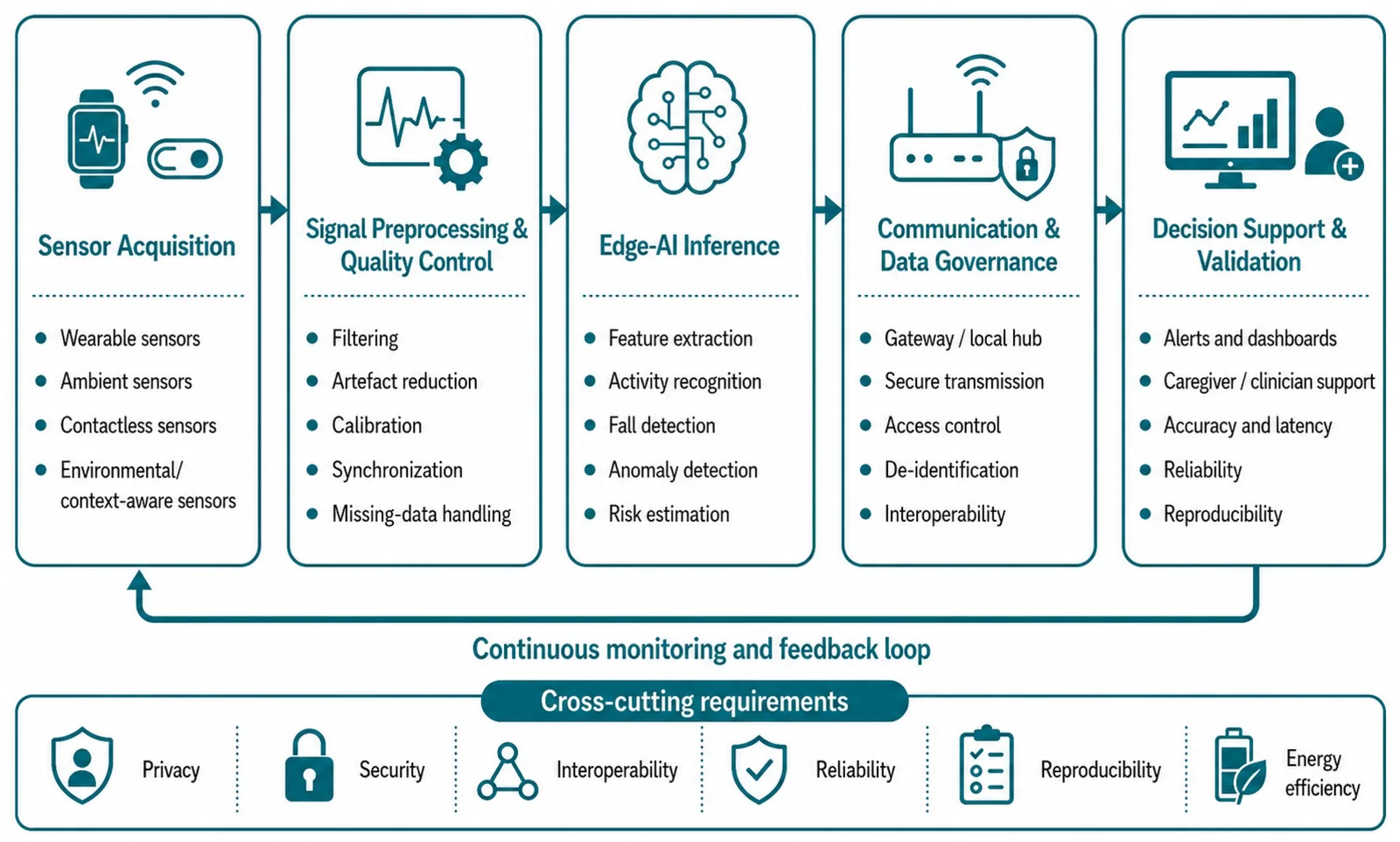
Proposed Edge-AI instrumentation framework for multimodal biometric sensing in active aging environments.

Figure 2 summarizes the proposed Edge-AI instrumentation framework. It shows how multimodal sensor acquisition, signal preprocessing and quality control, local inference, data governance, and decision support are connected within a continuous monitoring pipeline, while cross-cutting requirements such as privacy, security, interoperability, reliability, reproducibility, and energy efficiency operate across the full system.

The framework organizes the monitoring process into five interconnected layers: sensor acquisition, signal preprocessing and quality control, Edge-AI inference, communication and data governance, and decision support and validation. It highlights the need to treat active aging monitoring as a complete instrumentation pipeline in which privacy, security, interoperability, reliability, reproducibility, and energy efficiency must be considered across the full system.

Figure 2 should be interpreted as a conceptual framework rather than as an implemented or empirically validated architecture. The framework provides a structured basis for guiding the future design, comparison, and validation planning of Edge-AI monitoring systems in active aging environments. By linking sensor acquisition, signal quality control, local inference, data governance, decision support, and validation, it shows that reliable monitoring depends on the coherence of the full instrumentation pipeline, rather than on the isolated performance of individual sensors or algorithms.

### 4.4. Implications for Research and Practice

For research, the main implication is that future studies should report more than model performance. They should describe the full measurement and processing pipeline, including sensor placement, sampling conditions, calibration, preprocessing, quality control, synchronization, validation protocols, latency, energy use, privacy and security measures, and reproducibility details. This is particularly important for Sensors, where the value of a contribution depends not only on the novelty of a device or algorithm but also on the quality, transparency, and reproducibility of the sensing process.

For practice, the proposed framework can support the development of systems that are better aligned with real-world active aging environments. Older adults may live in homes with different layouts, routines, mobility levels, digital literacy, and privacy expectations. Care providers may require reliable alerts, interpretable indicators, and low-maintenance technologies. A framework that connects sensing, quality control, Edge AI, data governance, privacy, and validation can help developers design systems that are technically stronger and more suitable for long-term use.

Interoperability and data governance should also be considered in relation to established health-data and IoT standards. In sensor-based active aging environments, relevant directions include interoperable data models, standardized communication protocols, secure authentication mechanisms, and health-data exchange standards. Although the proposed framework does not prescribe a specific standard, it highlights the need to align future implementations with established interoperability and data-governance approaches, particularly when monitoring systems interact with clinical, caregiver, or institutional platforms.

The findings also have implications for standardization and comparison. Without common reporting dimensions, studies remain difficult to compare. A shared instrumentation-oriented structure can help researchers describe systems in a more consistent way, making it easier to assess which technologies are reliable, which are context-dependent, and which require further validation before real-world deployment.

### 4.5. Application Scenario: Fall-Risk and Abnormal-Inactivity Monitoring

To illustrate how the proposed framework can be applied, a home-based fall-risk and abnormal-inactivity monitoring scenario can be considered. In this scenario, the sensor acquisition layer combines wearable inertial sensors, such as accelerometers or inertial measurement units, with ambient sensors, such as passive infrared sensors, pressure sensors, door sensors, or room-level motion detectors. The purpose is not only to detect a fall event but also to monitor changes in mobility, inactivity, room transitions, and daily activity patterns that may indicate increased vulnerability in an older adult.

In the signal preprocessing and quality-control layer, wearable data would be filtered to reduce motion artifacts, segmented into relevant time windows, and checked for missing values, battery-related interruptions, and inconsistent device placement. Ambient data would be cleaned to reduce noise from room-layout constraints, sensor occlusion, or multi-occupant ambiguity. Synchronization between wearable and ambient data would be required to determine whether a sudden acceleration, lack of movement, or prolonged inactivity corresponds to a real risk event or to a normal daily activity.

In the Edge-AI inference layer, local models could classify movement patterns, identify possible falls, detect abnormal inactivity, or estimate short-term fall risk based on deviations from the older adult’s usual baseline. In this context, thresholds should not be defined only from generic adult populations. They should be adjusted according to individual mobility patterns, frailty level, assistive-device use, living environment, and prior activity history. This is particularly important because false negatives may delay assistance after a fall or risk event, whereas false positives may increase alarm fatigue and reduce user trust.

In the communication and data-governance layer, only selected alerts, indicators, or summarized risk information should be transmitted to caregivers, clinicians, or monitoring platforms. Raw biometric or behavioral data should be minimized whenever possible, and communication should follow privacy-aware and secure data-governance rules. In active aging environments, this is important because monitoring should support safety while preserving autonomy, dignity, and privacy.

In the decision-support and validation layer, the system output could be translated into alerts, risk indicators, or dashboard information for caregivers or health professionals. Validation should not be limited to classification accuracy. It should also include latency, missed events, false alarms, usability, user adherence, battery autonomy, interoperability with care platforms, privacy preservation, and reproducibility of the sensing and processing pipeline. Finally, the feedback loop would allow sensor recalibration, threshold adjustment, model updating, and alert refinement based on validation results, caregiver feedback, user experience, and changes in the older adult’s functional condition over time.

This scenario shows how the proposed framework can be used as a practical guide for designing, reporting, and validating active aging monitoring systems. It also illustrates how the framework helps identify gaps in existing studies, particularly when sensor placement, preprocessing, synchronization, threshold definition, latency, data governance, or validation criteria are not sufficiently reported.

### 4.6. Limitations

This study has some limitations. First, the corpus is strongly concentrated in recent years and includes a high proportion of studies from Sensors. This is justified by the focus of the article on sensor-based instrumentation, wearable and ambient sensors, and Edge-AI-enabled monitoring, but it may also influence the thematic distribution of the results. To reduce this limitation, the search included multidisciplinary and publisher-based databases, as well as studies from IEEE, JMIR, Frontiers, Elsevier, Springer-related sources, and other venues.

A further limitation is that formal inter-rater agreement statistics, such as Cohen’s kappa, were not calculated for the screening and eligibility stages. However, disagreements and uncertain cases were resolved through discussion among the authors until consensus was reached.

Another limitation is that the categories used in the results are not mutually exclusive. Several studies addressed more than one sensor modality, application context, or processing architecture. This reflects the nature of the field but also means that the numbers should be interpreted as thematic frequencies rather than exclusive classifications.

Third, the study did not perform a statistical meta-analysis because the included studies were heterogeneous in sensor type, target population, setting, architecture, validation method, and outcome measures. A descriptive and thematic synthesis was therefore more appropriate for the aims of this paper.

Fourth, the proposed framework is conceptual, evidence-informed, and instrumentation-oriented. It is grounded in the included literature, but it has not yet been empirically validated through prototype implementation or real-world deployment. Therefore, the framework should be interpreted as a structured design and validation guide rather than as a validated operational architecture. Future work should test the framework through prototype development, pilot studies, real-world deployments, and comparative validation across different sensing configurations.

## 5. Conclusions

This article proposed an Edge-AI instrumentation framework for multimodal biometric sensing in active aging environments. The study was supported by a structured analysis of 38 studies published between 2021 and 2025, selected from an initial database of 110 candidate records and retained after duplicate removal, screening, full-text eligibility assessment, and final reference cross-checking.

The findings show that active aging monitoring is a rapidly expanding research area. Most studies were published between 2023 and 2025, confirming the recent growth of sensor-based and AI-supported approaches in this field. The study also showed that movement-related monitoring remains dominant, especially in fall detection, gait analysis, activity recognition, mobility assessment, and ADL monitoring. Wearable sensors are widely used because they provide direct information about body movement and functional status, while ambient and contactless sensors offer less intrusive ways to monitor routines, inactivity, room transitions, and environmental context.

Despite these advances, the analysis identified important gaps. Many studies remain focused on isolated devices, specific applications, or AI classification results, while fewer studies describe the complete instrumentation process needed for real-world use. Signal quality, calibration, synchronization, latency, energy consumption, interoperability, privacy, security, and reproducibility remain critical challenges. These issues determine whether sensor-based systems can be trusted, compared, validated, replicated, and deployed in daily living environments.

The proposed framework addresses these gaps by organizing active aging monitoring as a structured instrumentation pipeline. It connects sensor acquisition, signal preprocessing and quality control, Edge-AI inference, communication and data governance, and decision support and validation. This structure can help researchers and developers plan, design, compare, and validate future monitoring systems with greater attention to reliability, privacy, interpretability, and reproducibility.

The main contribution of this article is therefore twofold. First, it provides a structured synthesis of recent research on multimodal sensing and Edge-AI-supported monitoring for active aging. Second, it proposes a conceptual and instrumentation-oriented framework that can guide future research, prototype development, and real-world validation. Future work should apply and test the framework in practical environments, including private homes, assisted-living facilities, rehabilitation contexts, and community-based active aging programs. Because the framework has not yet been empirically validated, future work should implement and test it in real active aging environments to assess its technical feasibility, usability, reliability, scalability, and practical value.

## Figures and Tables

**Table 1:** Database/source-level distribution of candidate records retained after filtering.

Source	Search Date	Records Retained After Filters, *n* (%)
MDPI/Sensors journal-specific search	25 April 2026	85 (77.3%)
IEEE Xplore	25 April 2026	2 (1.8%)
PubMed/JMIR	25 April 2026	7 (6.4%)
ScienceDirect/Elsevier	25 April 2026	5 (4.5%)
SpringerLink/BMC/Frontiers	25 April 2026	7 (6.4%)
Scopus/Web of Science cross-source verification	25 April 2026	4 (3.6%)
Total		110 (100.0%)

**Table 2:** General characteristics of the included studies.

Dimension	Main Result
Included studies	38 (100.0%)
Publication year distribution	2025: 15 (39.5%); 2024: 13 (34.2%); 2023: 7 (18.4%); 2022: 1 (2.6%); 2021: 2 (5.3%)
Studies published in 2023–2025	35 studies (92.1%)
Studies from Sensors	26 studies (68.4%)
Studies from other sources	12 studies (31.6%)
Most common document type	Research article 26 (68.4%)
Reviews and review-like studies	10 (26.3%)
Pilot or validation-focused studies	2 (5.3%)

Note: Document-type categories were grouped from the detailed coding reported in Supplementary File S1. Reviews and review-like studies include systematic reviews, scoping reviews, review articles, and systematic review and meta-analysis studies. Pilot or validation-focused studies include pilot studies and validation studies.

**Table 3:** Quality assessment summary of the included studies.

Quality Assessment Criterion	Main Observation	Influence on Framework Development
Sensor type and sensing modality	Usually reported with sufficient detail	Supported the definition of the sensor acquisition layer
Sensor placement and acquisition conditions	Inconsistently reported across studies	Reinforced the need for explicit acquisition reporting requirements
Sampling frequency and preprocessing	Frequently incomplete or only partially described	Supported the signal preprocessing and quality-control layer
Calibration and synchronization	Weakly reported in many studies	Justified the inclusion of calibration and temporal-alignment requirements
AI or signal-processing method	Usually reported, especially in AI/ML studies	Supported the Edge-AI inference layer
Validation strategy and metrics	Often focused on accuracy or classification performance	Supported broader validation criteria in the decision-support and validation layer
Reproducibility details	Frequently limited	Reinforced reproducibility as a cross-cutting framework requirement

**Table 4:** Sensing modalities identified in the included studies.

Sensing Modality	Studies, *n* (%)	Main Examples
Motion, mobility, ADL, or fall-related sensing	34 (89.5%)	Fall detection, gait, balance, activity recognition, frailty, mobility loss
Wearable/body-worn sensing	25 (65.8%)	IMUs, accelerometers, gyroscopes, smart watches, wristbands, wearable motion sensors
Ambient/non-wearable sensing	16 (42.1%)	Binary sensors, passive sensors, smart-home sensors, pressure sensors, environmental sensors
Contactless/radar/vision/Wi-Fi sensing	7 (18.4%)	Radar, Wi-Fi sensing, depth cameras, vision-based systems, non-contact fall detection
Multimodal/sensor-fusion systems	4 (10.5%)	Wearable–ambient integration, multimodal IoT monitoring, hybrid sensing
Physiological sensing	1 (2.6%)	Heart-related, respiratory, temperature, or other physiological monitoring

**Table 5:** Main processing architectures and analytical approaches identified in the corpus.

Processing or Analytical Approach	Studies, *n* (%)	Interpretation
AI/ML-based analysis	29 (76.3%)	Strong use of classification, prediction, anomaly detection, and activity-recognition methods
Edge/local/embedded processing	4 (10.5%)	Directly relevant to low-latency and privacy-aware monitoring
IoT/cloud/smart-home architecture	7 (18.4%)	Supports integration and remote monitoring but may increase interoperability and privacy challenges
Signal/statistical/optimization processing	5 (13.2%)	Relevant for preprocessing, feature extraction, and signal-quality control
Processing not central or not clearly reported	1 (2.6%)	Indicates limited technical reproducibility in part of the literature

**Table 6:** Application contexts identified in the included studies.

Application Context	Studies, *n* (%)	Main Focus
Older adults/active aging	24 (63.2%)	Autonomy, safety, independent living, functional monitoring
Health monitoring/care support	12 (31.6%)	Home monitoring, remote monitoring, caregiver support, rehabilitation
Fall detection/fall-risk assessment	13 (34.2%)	Fall events, fall prediction, risk classification, emergency response
Activity recognition/ADL monitoring	16 (42.1%)	Daily routines, movement patterns, behavioral monitoring
Mobility, gait, balance, or frailty	10 (26.3%)	Functional decline, gait analysis, balance, frailty assessment
Smart home/assisted living	12 (31.6%)	Ambient monitoring, home-based sensing, assisted-living environments

**Table 7:** Main instrumentation and validation challenges identified in the structured analysis.

Challenge	Studies in Which the Challenge Was Identified, *n* (%)	Relevance to Active Aging Monitoring
Signal quality	38 (100.0%)	Determines whether sensor outputs can support reliable interpretation under real-world conditions
Calibration	8 (21.1%)	Supports measurement consistency across devices, users, environments, and time
Synchronization	15 (39.5%)	Enables reliable integration of physiological, motion-related, behavioral, and environmental data
Latency	16 (42.1%)	Affects timely response in falls, inactivity, and risk-related events
Energy consumption	28 (73.7%)	Determines feasibility of long-term wearable and edge-device deployment
Interoperability	35 (92.1%)	Affects integration across sensors, platforms, gateways, and care systems
Privacy and security	16 (42.1%)	Protects sensitive biometric and behavioral information
Reproducibility	17 (44.7%)	Determines whether systems can be replicated, compared, validated, and extended

Note: Counts refer to studies in which each challenge was identified as relevant to system design, implementation, validation, deployment, or reproducibility. They do not indicate that the challenge was fully addressed or resolved by the study.

**Table 8:** Mapping between research questions and main synthesis results.

Research Question	Main Synthesis Result	Study-Level Evidence from the Final Synthesis Corpus
RQ1. What types of wearable, ambient, and context-aware sensors are used for biometric and functional monitoring in active aging environments?	The corpus is dominated by motion, mobility, ADL, and fall-related sensing, with strong use of wearable/body-worn technologies and a smaller but relevant presence of ambient, contactless, and multimodal systems.	Motion, mobility, ADL, or fall-related sensing: 34 studies (89.5%); wearable/body-worn sensing: 25 studies (65.8%); ambient/non-wearable sensing: 16 studies (42.1%); contactless/radar/vision/Wi-Fi sensing: 7 studies (18.4%); multimodal/sensor-fusion systems: 4 studies (10.5%).
RQ2. Which physiological, motion-related, behavioral, and environmental signals are most frequently collected and analyzed in these systems?	Based on the modality and application-context coding used as a proxy for measured signal groups, motion-related and functional signals were the most frequent, followed by behavioral/ADL-related and environmental/contextual signals. Physiological signals were much less represented in the final synthesis corpus.	Motion-related and functional signals, including fall, gait, balance, mobility, frailty, posture, and activity-related indicators: 34 studies (89.5%); behavioral and ADL-related signals, including routines, inactivity, daily activity patterns, and room transitions: 16 studies (42.1%); environmental/contextual signals, including ambient, smart-home, pressure, room-level, and proximity/contextual information: 16 studies (42.1%); primarily physiological signals, including heart-related, respiratory, temperature, or oxygen-saturation indicators: 1 study (2.6%).
RQ3. How are Edge AI, edge computing, or local processing approaches used to support real-time monitoring, anomaly detection, and decision support?	AI/ML-based analysis was frequent, but explicit Edge/local/embedded processing was less common. This indicates that many studies still emphasize algorithmic classification more than complete Edge-AI instrumentation pipelines.	AI/ML-based analysis: 29 studies (76.3%); Edge/local/embedded processing: 4 studies (10.5%); IoT/cloud/smart-home architecture: 7 studies (18.4%); signal/statistical/optimization processing: 5 studies (13.2%); processing not central or not clearly reported: 1 study (2.6%).
RQ4. What instrumentation challenges are reported in relation to signal quality, calibration, synchronization, latency, energy consumption, reliability, interoperability, privacy, and reproducibility?	The main instrumentation challenges were signal quality, interoperability, energy consumption, reproducibility, latency, privacy/security, synchronization, and calibration. These counts indicate that the challenges were identified as relevant, not necessarily that they were fully addressed or solved.	Signal quality: 38 studies (100.0%); interoperability: 35 studies (92.1%); energy consumption: 28 studies (73.7%); reproducibility: 17 studies (44.7%); latency: 16 studies (42.1%); privacy and security: 16 studies (42.1%); synchronization: 15 studies (39.5%); calibration: 8 studies (21.1%).
RQ5. Which validation criteria are used to assess the technical and practical performance of sensor-based systems for active aging?	Validation was most commonly associated with performance metrics and technical feasibility, but broader criteria such as latency, energy consumption, privacy/security, interoperability, reproducibility, usability, and long-term deployment readiness were less consistently reported.	Validation strategy or performance metrics were fully reported in 28 studies (73.7%) and partially reported in 10 studies (26.3%). Additional validation-related criteria identified across the corpus included signal quality/reliability, latency, energy consumption, privacy/security, interoperability, reproducibility, calibration, synchronization, and practical deployment constraints.

**Table 9:** Relationship between literature findings, framework layers, and design implications.

Literature Finding	Framework Layer	Design Implication
Inconsistent reporting of sensor placement, acquisition conditions, and sampling parameters	Sensor acquisition layer	Requires explicit reporting of sensor type, placement, sampling frequency, acquisition environment, calibration requirements, and expected sources of noise
Limited description of preprocessing, signal-quality assessment, artifact reduction, calibration, and synchronization	Signal preprocessing and quality-control layer	Requires filtering, segmentation, normalization, missing-data handling, artifact reduction, calibration, and temporal alignment across heterogeneous data streams
Frequent use of AI/ML methods, but limited implementation of complete Edge-AI pipelines	Edge-AI inference layer	Requires local feature extraction, classification, anomaly detection, risk estimation, computational-efficiency criteria, inference stability, and update mechanisms
Recurrent concerns about privacy, interoperability, data transfer, and long-term system integration	Communication and data-governance layer	Requires secure communication, access control, data minimization, de-identification, interoperability, and privacy-aware data management
Validation often focused on accuracy, with less attention to usability, latency, energy use, reliability, and reproducibility	Decision-support and validation layer	Requires broader validation criteria, including accuracy, latency, reliability, energy consumption, usability, privacy, security, interoperability, reproducibility, and real-world interpretability
Need for long-term adaptation in changing home and assisted-living environments	Continuous monitoring and feedback loop	Requires alert tuning, system recalibration, preprocessing refinement, model updating, user feedback integration, and repeated validation over time

**Table 10:** Comparison between existing monitoring approaches and the proposed Edge-AI instrumentation framework.

Existing Approach	Main Emphasis	Limitation for Active Aging Instrumentation	Contribution of the Proposed Framework
IoT-based elderly monitoring frameworks	Connectivity, data collection, remote monitoring, and integration with external platforms	Often focus on data transmission and system connectivity, with limited attention to calibration, measurement uncertainty, signal quality, and validation	Adds instrumentation-oriented acquisition, calibration, signal-quality control, synchronization, and validation requirements
Edge-intelligence monitoring frameworks	Local processing, latency reduction, and reduced dependence on cloud infrastructures	Frequently emphasize inference or anomaly detection, but do not always describe the full measurement and preprocessing pipeline	Integrates Edge AI with signal preprocessing, quality control, computational constraints, data governance, and validation
Wearable-based fall detection or activity-recognition systems	Device-based monitoring, movement classification, fall detection, and activity recognition	Often application-specific, device-dependent, and focused mainly on classification performance	Places fall detection and activity recognition within a broader multimodal monitoring pipeline
Smart-home and AAL architectures	Ambient monitoring, home integration, routine detection, and assisted-living support	May be limited by interoperability, privacy, multi-occupant ambiguity, and weak individual-level physiological interpretation	Adds cross-cutting requirements for interoperability, privacy, security, reproducibility, reliability, and energy efficiency
Proposed Edge-AI instrumentation framework	Integrated instrumentation pipeline for multimodal Edge-AI monitoring in active aging environments	Conceptual and evidence-informed; requires prototype implementation and empirical validation	Provides an integrated structure linking sensor acquisition, signal quality control, calibration, synchronization, local inference, data governance, decision support, feedback, and validation

## Data Availability

The data supporting the findings of this study are included in the article and in Supplementary File S1. The Supplementary File contains the search log, screening decisions, PRISMA counts, exclusion reasons, quality assessment scores, and the final data extraction table.

## Supplementary Materials

The following supporting information can be downloaded at: https://www.mdpi.com/article/10.3390/s26165072/s1, Supplementary File S1: Structured literature analysis database and PRISMA 2020 documentation.

## Abbreviations

The following abbreviations are used in this manuscript:

ADL	Activities of Daily Living
AI	Artificial Intelligence
AI/ML	Artificial Intelligence/Machine Learning
IMU	Inertial Measurement Unit
IoT	Internet of Things
ML	Machine Learning
PRISMA	Preferred Reporting Items for Systematic Reviews and Meta-Analyses
Wi-Fi	Wireless Fidelity
